# Novel Microbial-Based Immunotherapy Approach for Crohn's Disease

**DOI:** 10.3389/fmed.2019.00170

**Published:** 2019-07-19

**Authors:** Simon Sutcliffe, Shirin Kalyan, Jim Pankovich, Jenny M. H. Chen, Rashieda Gluck, Darby Thompson, Momir Bosiljcic, Mark Bazett, Richard N. Fedorak, Remo Panaccione, Jeffrey Axler, John K. Marshall, David W. Mullins, Boyko Kabakchiev, Dermot P. B. McGovern, Julie Jang, Andrew Coldman, Gillian Vandermeirsch, Brian Bressler, Hal Gunn

**Affiliations:** ^1^Qu Biologics Inc., Vancouver, BC, Canada; ^2^Department of Medicine, University of British Columbia, Vancouver, BC, Canada; ^3^Emmes Canada, Burnaby, BC, Canada; ^4^Department of Statistics and Actuarial Sciences, Simon Fraser University, Burnaby, BC, Canada; ^5^Division of Gastroenterology, University of Alberta, Edmonton, AB, Canada; ^6^Inflammatory Bowel Disease Unit, University of Calgary, Calgary, AB, Canada; ^7^Toronto Digestive Disease Associates Inc., Vaughan, ON, Canada; ^8^Department of Medicine and Farncombe Family Digestive Health Research Institute, McMaster University, Hamilton, ON, Canada; ^9^Department of Microbiology, Immunology and Medical Education, Geisel School of Medicine at Dartmouth, Hanover, NH, United States; ^10^Zane Cohen Centre for Digestive Diseases, Samuel Lunenfeld Research Institute, Mount Sinai Hospital, Toronto, ON, Canada; ^11^Cedars-Sinai Medical Center, Los Angeles, CA, United States; ^12^Cancer Control Research, British Columbia Cancer Agency, Vancouver, BC, Canada; ^13^Gastrointestinal Research Institute, Vancouver, BC, Canada

**Keywords:** Crohn's disease, randomized placebo-controlled trial, immunotherapy, innate immunity, biologic, biomarkers, microbial-based therapy

## Abstract

**Background:** Current Crohn's disease (CD) therapies focus on suppressing immune function and come with consequent risk, such as infection and cancer. Notwithstanding, most CD patients still experience disease progression. There is a need for new CD treatment strategies that offer better health outcomes for patients.

**Aims:** To assess safety, efficacy, and tolerability of a novel microbial-derived immunotherapy, QBECO, that aims to restore rather than suppress immune function in CD.

**Methods:** A randomized, double-blind, placebo-controlled trial was conducted in 68 patients with moderate-to-severe CD. Primary endpoints: safety and Week 8 clinical improvement. Secondary endpoints: Week 8 clinical response and remission. Week 8 responders continued blinded treatment through Week 16; non-responders received open-label QBECO from Weeks 9–16. Exploratory analyses included immune biomarker and genotype assessments.

**Results:** QBECO was well-tolerated. Mean reduction in Crohn's Disease Activity Index (CDAI) score was −68 for QBECO vs. −31 for placebo at Week 8. Improvement with QBECO continued through Week 16 (-130 CDAI reduction). Week 8 QBECO clinical response, improvement and remission rates were 41.2%, 32.4%, 29.4% vs. 26.5%, 23.5%, 23.5% for placebo. TNFα inhibitor-naïve subjects achieved higher response rates at Week 8 with QBECO (64%) vs. placebo (26%). Specific immune biomarkers were identified that linked to QBECO response.

**Conclusion:** This proof-of-concept study supports further investigation for the use of QBECO as a novel immunotherapy approach for CD. Biomarker analyses suggests it may be feasible to personalize CD treatment with QBECO. Larger trials are now needed to confirm clinical improvement and the unique biological findings.

**Clinical Trial Number:** NCT01809275 (https://clinicaltrials.gov/ct2/show/NCT01809275)

## Introduction

Crohn's disease (CD) is a chronic inflammatory disease of the gastrointestinal (GI) tract with a relapsing and remitting course. Chronic uncontrolled inflammation can lead to progressive bowel damage and complications such as stenosis and fistula, often leading to surgery (1, 2). Front-line therapies for CD target adaptive immune pathways, and many patients treated with these immunosuppressive therapeutics still develop progressive disabling disease (2) as well as increased risk of infections, malignancy, lupus-like autoimmunity, demyelinating central nervous system disease, and hypersensitivity reactions (3, 4). Thus, new treatment approaches for CD that are safe, cost-effective, and able to achieve durable remission are required.

Accumulating clinical and genetic evidence suggests that a defective innate immune response may be fundamental to the pathogenesis of CD (5–8) and precedes the consequent over-reactive adaptive immune response that is characteristic of the disease and the target of current treatments (9, 10). Segal and Lowei first demonstrated that patients with CD exhibited an impaired systemic acute inflammatory response (11). Subsequent genetic studies provided further support for the hypothesis that defective or inefficient innate immune function, particularly that of macrophages, is linked to CD (12, 13). In this work, we present the results from a Phase 1/2 randomized, double-blind, placebo-controlled trial of a novel immunotherapy approach to optimize innate immune function in CD. QBECO, an investigational immunotherapy derived from an inactivated GI pathogen, aims to elicit an acute innate immune response targeting the GI tract to re-establish competent barrier function and immune competency (14). Treatment is self-administered by subcutaneous injection. Promising early clinical experience with QBECO for the treatment of CD in a compassionate use program (15) and a translational study in ulcerative colitis showing improved GI barrier function with QBECO treatment (14) motivated this proof-of-concept clinical trial to explore safety, efficacy, and tolerability of this novel immunotherapy in subjects with moderate-to-severe CD.

## Methods

### Study Design, Randomization, and Treatment Strategy

This was a Phase 1/2 randomized, double-blind, placebo-controlled study (NCT01809275; Health Canada approval 27-02-2013) for the treatment of moderate-to-severe CD. All study subjects provided written informed consent and the trial was conducted in compliance with the Declaration of Helsinki and Good Clinical Practice guidelines. The study protocol was approved by the institutional research ethics boards at the four study sites.

Eligible subjects were randomized 1:1 to receive self-administered subcutaneous injections of blinded placebo or blinded QBECO every second day for 8 weeks. At the time the study was conducted, the Crohn's Disease Activity Index (CDAI) was the instrument of choice to assess disease severity (16–18). The CDAI is composed of 8 weighted components present over 7 days which include: number of liquid/soft stools per day, abdominal pain, general feeling of well-being, presence of complications, taking opioid-based medication for diarrhea, presence of an abdominal mass, hematocrit <0.47 for men or <0.42 in women, and deviation from one's normal weight (by percentage). Subjects with a clinical response (defined as a decrease in CDAI ≥ 70 points) at Week 8 continued their randomized assignment in a blinded manner through 16 weeks. Subjects without a clinical response at Week 8 received open-label QBECO from Week 9–16. Subcutaneous injection procedures were identical for the randomized groups and included an initial 5-day training at the study site. Subjects titrated their dose beginning at 0.05 mL, increasing by 0.02 mL every other day until experiencing a 2.5–5 cm erythema at the injection site on the day following injection, or until a maximum 0.2 mL dose was reached. The dose was reduced by 0.01 mL if erythema exceeded 5 cm until the targeted 2.5–5 cm erythema was reached. Subjects switching to open-label QBECO at 8 weeks were re-titrated irrespective of their randomized group. Randomization was based on a pre-defined list using a permuted block design (size 4). Product was dispensed in randomly numbered kits. Pharmacy, clinical site staff and subjects were blinded to assignment.

### Patient Population

Subjects ≥ 18 years with a diagnosis of CD of >6 months duration established by clinical, endoscopic or radiological, and histopathological assessment were enrolled following informed consent. All subjects had moderate-to-severe disease, defined by CDAI > 220 but <450 points, AND either a CRP (C-reactive protein) level > 2.87 mg/L or a fecal calprotectin (FCP) level > 250 μg/g, or an ileocolonoscopy or radiographic tests showing active CD within the last 6 months. CRP, FCP, and standard laboratory measures were taken at each study visit. Continued stable doses of the following medications were allowed: oral 5-ASA compounds, oral corticosteroid prednisone equivalent dose <30 mg/day or budesonide <9 mg/day if there was dose stability >2 weeks before trial screening visit; probiotics, anti-diarrheal medications, azathioprine or 6-mercaptopurine, or methotrexate provided the dose had been stable for 8 weeks preceding first study dose; and antibiotics providing dose stability was present for 2 weeks prior to first study dose. Male and pre-menopausal female subjects were required to agree to practice effective birth control.

### QBECO and Placebo Composition, Formulation, and Administration

QBECO is an investigational immune modulator consisting of all major macromolecules of an inactivated pathogenic strain of *E. coli*, isolated from a patient with an *E. coli* GI infection, suspended in physiological saline with 0.4% phenol as a preservative. The placebo used in this clinical trial was physiological saline with 0.4% phenol. Study ampules were tinted to prevent comparison of turbidity between QBECO and placebo.

Following three in-clinic supervised doses, subjects self-administered QBECO at home, recording the date of the injection, dose (volume) injected, location of the injection site, local skin response diameter (if present) on the day following administration, and any other relevant observations. Compliance was evaluated by diary review and clinic visit assessment.

### Safety Assessment

Safety variables included adverse events, concomitant therapies, physical exams, and laboratory tests to assess hematologic, hepatic, and renal function at scheduled time points. Adverse events were graded according to National Cancer Institute Common Terminology Criteria, Version 4.0 (http://avs.nci.nih.gov). Subjects experiencing serious adverse events discontinued study treatment.

### Study Outcome Variables

Primary endpoints were safety (adverse events, clinical laboratory findings, concomitant therapies) and clinical improvement defined as a decrease in CDAI from baseline of ≥100 points or a CDAI score of ≤ 150 points at Week 8. Secondary endpoints were clinical remission at Week 8 (CDAI ≤ 150 points); clinical response at Week 8 (decrease in CDAI score from baseline ≥ 70 points); and CDAI score change from baseline to Week 8. Treatment failure was defined by the need for rescue medications, major surgical intervention for the treatment of CD, or QBECO-related adverse events leading to discontinuation of QBECO.

Exploratory end-points included the relationship between QBECO response and baseline demographic and disease characteristics, concomitant therapies, serum immune biomarkers, and CD-associated genotypes.

### Immune Biomarker Analysis

Serum immune cytokines and chemokines were analyzed by multiplex technology (assay performed by Eve Technologies, Calgary, AB, Canada) using the Human Cytokine/Chemokine Array 42-Plex with IL-18 (HD42; Millipore).

### Genetic Analysis

Genetic analysis with respect to QBECO response/non-response was performed on 30 CD subjects treated with QBECO, including 27 subjects from this trial and 3 subjects treated in a compassionate use program (15). Genetic analysis was not included in the original trial design and thus, consent for genetic testing sought retrospectively in subjects treated with QBECO. The analysis was done on 16 trial subjects who provided consent, 11 trial subjects for whom ethics approval was obtained for genome analysis on anonymized samples identified only as “QBECO responder” or “QBECO non-responder,” and 3 subjects treated in a compassionate use program who provided consent.

Genotyping was performed using the InfiniumOmni2-5-8 v1.3 array (Illumina, San Diego, CA). Preprocessing and quality control were completed in Genome Studio v2011.1 (Illumina). Only single-nucleotide polymorphisms (SNPs) with GenCall scores >0.2 in 90% of samples, call rates > 95%, minor allele frequency >5%, and Hardy–Weinberg equilibrium *p*-values > 10^−6^ were considered for further analysis. Two-hundred forty IBD susceptibility SNPs (19, 20) were selected in a hypothesis-driven approach; 113 passed quality control for analysis.

### Statistical Analysis

An initial sample size of 60 subjects was chosen to achieve 80% power with a two-sided 0.05-level chi-square test assuming a 42% (33 vs. 75%) difference in clinical improvement rate between QBECO and placebo at Week 8. An interim sample-size re-estimation was performed based on aggregate, blinded, clinical improvement rates, and the sample size was increased to 68 subjects.

Week 8 and 16 clinical response, improvement and remission rates were compared between placebo and QBECO groups using a 2-sided Fisher's Exact test. Change in CDAI from baseline was compared using a 2-sided, 2-sample paired *t*-test.

**Figure 1:**
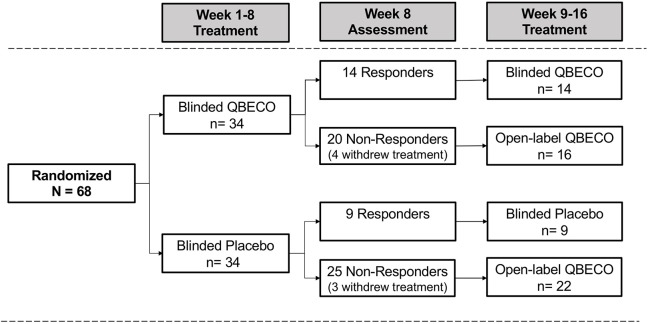
Patient flow by treatment assignment. Subjects with moderate-to-severe Crohn's disease were randomized 1:1 to QBECO or Placebo for 8 weeks. Those responding to allocated blinded treatment continued blinded treatment for another 8 weeks; all 8 Week non-responders commenced open-label QBECO for 8 weeks.

The intention to treat (ITT) analysis evaluated all 68 subjects by randomized group. Subjects considered as treatment failures or who did not provide Week 8 CDAI scores were considered non-responders in the ITT analysis. The Week 8 per protocol (PP) analysis excludes 10 subjects who did not complete 8 weeks of treatment (*n* = 8) or failed to meet all eligibility criteria (*n* = 2), comprised of 7 from the QBECO arm and 3 from the placebo arm (Figure 1).

To account for imbalances in important baseline variables, change in CDAI score from baseline through Week 8 was modeled using a linear regression model that included the following criteria: CDAI, disease severity at baseline (≥250 vs. <250), use of concomitant immunosuppressive medication (Y/N), disease duration (years) and prior exposure to anti-TNF agents (Y/N). These variables were selected prior to the regression analysis based on scientific and clinical judgement; no model selection was performed. Population marginal means for QBECO and placebo (i.e., assuming a balanced population) were generated using least-square means analysis and statistically compared using a *t*-test.

Cytokines were evaluated by change in concentration over time with exposure to study treatment (using one-sample Mann-Whitney test of the paired differences) and by differential change over time by QBECO clinical response status (in QBECO treated subjects only). Linear mixed effect model of cytokine concentration was computed with *p*-values estimated from a Wald test of the interaction between clinical response and time. Finally, associations between baseline concentration of cytokines and Week 8 clinical response and remission status (two-sample Mann-Whitney) were assessed. *P*-values were adjusted for multiple comparisons across 42 cytokines using a Benjamini-Hochberg procedure.

Genetic analysis used generalized linear models with an additive genetic model for categorical response and predictor variables. Kruskal-Wallis analysis of variance was used for continuous and ordinal response variables. Benjamini-Hochberg procedure was used to adjust for multiple comparisons across the 113 SNPs that passed quality control. An algorithm combining the top three SNPs linked to QBECO response was formulated using methodology previously described in a genetic association study of CD and ulcerative colitis phenotypes (21).

All authors had access to the study data and reviewed and approved the final manuscript.

## Results

### Study Subjects

Sixty-eight subjects were randomly assigned (1:1) to receive blinded QBECO or placebo for 8 weeks. Demographic and baseline characteristics are shown for both treatment groups in Table 1. The two arms markedly differed with respect to prior anti-TNFα exposure (59% QBECO vs. 21% placebo). Patients randomized to QBECO were older (average age 42.4 vs. 31.8 years for placebo), and more likely to have baseline CDAI > 250 compared to the placebo group (24 vs. 19).

**Table 1:** Baseline and demographic variables.

**Variable**	**All (*n* = 68)**	**QBECO (*n* = 34)**	**Placebo (*n* = 34)**
**SEX**, ***n*** **(%)**			
Male	38 (55.9)	20 (58.8)	18 (52.9)
Female	30 (44.1)	14 (41.2)	16 (47.1)
**RACE**, ***n*** **(%)**			
Caucasian	59 (86.8)	30 (88.2)	29 (85.3)
Black	1 (1.5)	0 (0.0)	1 (2.9)
Hispanic	2 (2.9)	2 (5.9)	0 (0.0)
Asian	5 (7.4)	2 (5.9)	3 (8.8)
Other	1 (1.5)	0 (0.0)	1 (2.9)
**AGE**			
Median (IQR)	36.1 (27.5, 50.5)	42.4 (30.6, 50.6)	31.8 (26.3, 47.7)
Mean (SD)	39.2 (14.0)	41.0 (12.3)	37.5 (15.4)
Range	(19.2, 80.1)	(21.0, 72.7)	(19.2, 80.1)
**YEARS SINCE INITIAL CD DIAGNOSIS^a^**			
Median (IQR)	7.9 (3.7, 13.0)	9.2 (3.8, 13.2)	6.0 (3.7, 10.9)
Mean (SD)	9.8 (8.4)	10.2 (7.6)	9.3 (9.2)
Range	(0.5, 49.3)	(0.5, 30.4)	(0.8, 49.3)
**AGE AT INITIAL CD DIAGNOSIS^a^**			
Median (IQR)	28.0 (20.3, 36.0)	30.0 (20.2, 39.2)	24.7 (20.3, 31.4)
Mean (SD)	29.5 (12.7)	30.8 (12.8)	28.1 (12.6)
Range	(9.6, 70.0)	(11.9, 70.0)	(9.6, 61.9)
**CDAI**			
Median (IQR)	265.0 (238, 328)	268.0 (241, 331)	260.0 (233, 323)
Mean (SD)	288.4 (64.4)	290.7 (57.1)	286.0 (71.7)
Range	(210.0, 449.0)	(210.0, 445.0)	(220.0, 449.0)
**BMI**			
Mean (SD)	25.0 (5.8)	25.1 (5.8)	24.8 (5.9)
Median (IQR)	24.3 (21.0, 28.4)	24.4 (20.5, 29.1)	24.1 (21.0, 28.1)
Range	(16.0, 40.3)	(16.4, 39.9)	(16.0, 40.3)
**FECAL CALPROTECTIN (ug/g)^b^**			
Median (IQR)	481.2 (258, 706)	450.0 (260, 641)	518.9 (242, 782)
Mean (SD)	574.7 (448.4)	523.2 (365.8)	626.2 (518.5)
Range	(15.6, 2000.0)	(15.6, 1519.5)	(24.9, 2000.0)
**C-REACTIVE PROTEIN (mg/L)**			
Median (IQR)	10.0 (4.4, 24.0)	8.5 (4.0, 21.4)	11.5 (5.4, 24.0)
Mean (SD)	17.4 (18.2)	15.8 (17.4)	18.9 (19.0)
Range	(0.1, 77.1)	(0.1, 59.5)	(3.1, 77.1)
**PRESENT OF FISTULAS**, ***n*** **(%)**			
Yes	8 (11.8)	4 (11.8)	4 (11.8)
No	36 (52.9)	17 (50.0)	19 (55.9)
Not done	24 (35.3)	13 (38.2)	11 (32.4)
Prior anti-TNFα treatment^b^, *n* (%)	27 (39.7)	20 (58.8)	7 (20.6)
Concomitant therapy for CD, *n* (%)	52 (76.5)	27 (79.4)	25 (73.5)
**TYPE OF CONCOMITANT THERAPY FOR CD^d^**, ***n*** **(%)**			
Aminosalicylates	12 (17.6)	7 (20.6)	5 (14.7)
Antibiotics	3 (4.4)	2 (5.9)	1 (2.9)
Anti-diarrheals	19 (27.9)	9 (26.5)	10 (29.4)
Corticosteroids	23 (33.8)	12 (35.3)	11 (32.4)
Immunomodulators	12 (17.6)	5 (14.7)	7 (20.6)
Others	5 (7.4)	4 (11.8)	1 (2.9)

a *For Crohn's diagnosis date, month was assumed to be July and day was assumed to be 15 if more exact information was not recorded*.

b *Values beyond the upper or lower detection limit were assigned the limit value for the calculation of summary statistics*.

c *Prior anti-TNF α was significantly different between groups, Fisher's Exact test p-value = 0.003*.

d *Multiple categories can be used for the same subject*.

*Others included iron infusion, Lyderm, probiotics, and VSL #3 probiotic*.

Overall, 56 of the 68 subjects (82.4%) completed the study through Week 24, including 27 (79.4%) subjects initially randomized to QBECO and 29 (85.3%) to placebo. Figure 1 shows the flow of subjects over 16 weeks by treatment. Compliance with treatment administration was high in both groups (>90% of expected injections).

### Change in Crohn's Disease Status by Treatment Group

**Figure 2:**
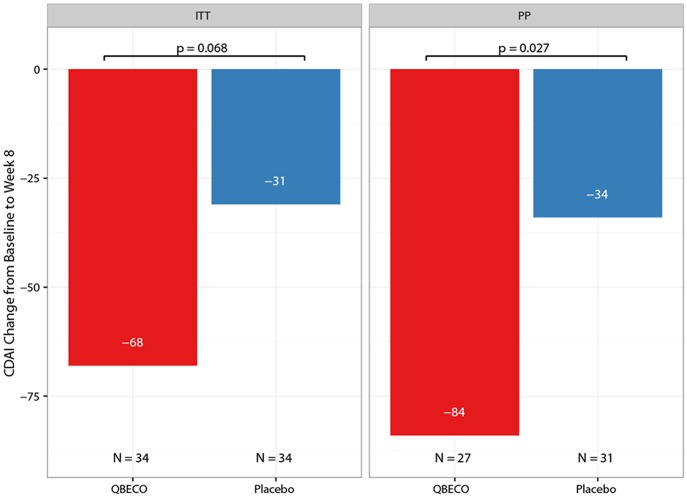
Mean change in Crohn's Disease Activity Index (CDAI) score from baseline to Week 8 by treatment group. The mean reduction in CDAI from baseline to Week 8 in patients with moderate-to-severe Crohn's disease blinded to QBECO (red bar) or Placebo (blue bar) treatment. ITT, Intention to Treat analysis; PP, Per Protocol analysis.

**Figure 3:**
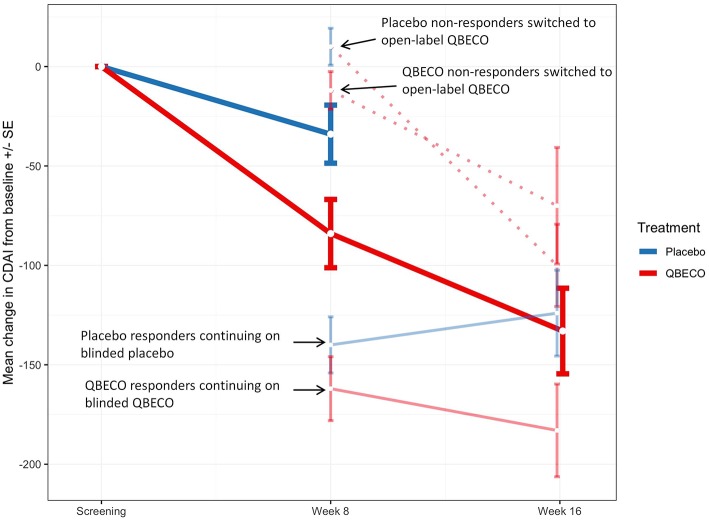
Mean change in Crohn's Disease Activity Index (CDAI) score in study groups Week 8 and Week 16. Mean change in CDAI score for subjects randomized to QBECO (solid red line) and placebo (solid blue line) from baseline to Week 8 and Weeks 16 of study treatment. Those responding to allocated blinded treatment at week 8 continued blinded treatment for another 8 weeks [light blue solid line for responders originally blinded to placebo treatment (*n* = 9); light red solid line for QBECO responders (*n* = 13)]. All placebo (*n* = 22) and QBECO (*n* = 14) non-responders at week 8 received open-label QBECO (dashed red lines) for weeks 9–16. Dark solid red line represents the average of all subjects on QBECO for weeks 9–16.

Mean reduction in CDAI score from baseline to Week 8 was greater for QBECO vs. placebo subjects in both the ITT (*p* = 0.068) and PP (*p* = 0.027) analyses (Figure 2). Table 2 presents the rates of clinical response (41.2% QBECO vs. 26.5% placebo), improvement (32.4% QBECO vs. 23.5% placebo) and remission (29.4% QBECO vs. 23.5% placebo), which did not achieve statistical significance at Week 8. Subjects treated with QBECO continued to improve through Week 16, experiencing a further mean CDAI reduction of 50 points from Week 8, reaching a 130-point reduction by Week 16 (Figure 3; Supplement Table 1 provides this data in tabular form, which includes the number of patients moving through each arm of the study to Week 16).

**Table 2:** Clinical response, improvement, and remission rates at Week 8 by treatment.

	**Intention to treat**			**Per protocol**		
**Outcome**	**QBECO *N* = 34**	**Placebo *N* = 34**	**% difference [95% CI]**	**QBECO *N* = 27**	**Placebo *N* = 31**	**% difference [95% CI]**
Response	14 (41.2%)	9 (26.5%)	14.7% [−10, 39]	13 (48.1%)	9 (29.0%)	19.1% [−7, 43]
Improvement	11 (32.4%)	8 (23.5%)	8.8% [−16, 33]	11 (40.7%)	8 (25.8%)	14.9% [−11, 39]
Remission	10 (29.4%)	8 (23.5%)	5.9% [−19, 30]	10 (37%)	8 (25.8%)	11.2% [−15, 36]
**Anti-TNFα** **naive**	***N*** **=** **14**	***N*** **=** **26**		***N*** **=** **14**	***N*** **=** **24**	
Response	9 (64.3%)	7 (26.9%)	37.4% [5, 65]	9 (64.3%)	7 (29.2%)	35.1% [2, 63]
Improvement	7 (50.0%)	6 (23.1%)	26.9% [−5, 57]	7 (50.0%)	6 (25.0%)	25.0% [−8, 55]
Remission	7 (50.0%)	6 (23.1%)	26.9% [−5, 57]	7 (50.0%)	6 (25.0%)	25.0% [−8, 55]

Patients naïve to anti-TNFα agents achieved a 64% response rate at Week 8 with QBECO vs. 26% placebo (*p* = 0.041; ITT analysis), and more than double the improvement and remission rates (Table 2). Anti-TNFα naïve Week 8 placebo non-responders (*n* = 17) treated with 8 weeks open-label QBECO from weeks 9 to 16 achieved response, improvement and remission rates of 71, 47, and 47%, respectively. This was similar to the response, improvement and remission rates observed after 8 weeks of treatment in the anti-TNFα naïve group initially randomized to QBECO. A longitudinal analysis including previous TNFα inhibitor exposure interaction with treatment following week 8 shows that subjects previously treated with anti-TNFα agents may respond to QBECO treatment with longer course of treatment (Supplement Figure 1).

In subjects with baseline CDAI ≥ 250 (*n* = 24 [70.6%] QBECO, *n* = 19 [55.9%] placebo), the response, improvement and remission rates in subjects treated with QBECO vs. placebo were: 42%, 29%, 25% vs. 16%, 11%, 11%, respectively. To account for differences in baseline characteristics that may be important in the change in CDAI score by treatment, a regression analysis was performed taking into account baseline CDAI, disease severity at baseline (>250), use of concomitant immunosuppressive medication, disease duration and prior exposure to anti-TNFα agents (Supplement Figure 2). Following adjustment for imbalanced prognostic variables, the reduction in CDAI at Week 8 was 48 points greater in the QBECO than placebo treated cohort (*p* = 0.024; ITT analysis).

#### Safety Evaluation

QBECO treatment was well-tolerated. Adverse events experienced by >5% of blinded participants for weeks 1–8 of the study (Table 3) and subjects who received open-label QBECO for weeks 9–16 (Table 4). The majority of adverse events reported during the blinded period (weeks 1–8) were Grade 1 (82.1 and 88.1% of all events for subjects receiving QBECO-01 and placebo, respectively) and transient. No significant difference between placebo and QBECO groups was identified. Those receiving QBECO experienced more transient flu-like symptoms, which are considered to be related to the mechanism of action of QBECO.

**Table 3:** Adverse events affecting at least 5% of subjects receiving study drug.

**System organ class**	**Number (%) of patients**		
**Preferred term**	**All (*n* = 68)**	**QBECO (*n* = 34)**	**Placebo (*n* = 34)**
**Weeks 1–8**			
Any Adverse Event	45 (66.2)	25 (73.5)	20 (58.8)
Common Adverse Events^*^			
Abdominal tenderness	3 (4.4)	1 (2.9)	2 (5.9)
Mouth ulceration	2 (2.9)	2 (5.9)	0 (0.0)
Nausea	8 (11.8)	6 (17.6)	2 (5.9)
Fatigue	8 (11.8)	4 (11.8)	4 (11.8)
Influenza like illness	14 (20.6)	10 (29.4)	4 (11.8)
Injection site bruising	2 (2.9)	0 (0.0)	2 (5.9)
Injection site pain	2 (2.9)	2 (5.9)	0 (0.0)
Injection site pruritus	5 (7.4)	5 (14.7)	0 (0.0)
Pyrexia	11 (16.2)	8 (23.5)	3 (8.8)
Dizziness	2 (2.9)	2 (5.9)	0 (0.0)
Headache	6 (8.8)	2 (5.9)	4 (11.8)
Cough	2 (2.9)	2 (5.9)	0 (0.0)
Oropharyngeal pain	2 (2.9)	2 (5.9)	0 (0.0)

* *Common adverse events were defined as events occurring in at least 5% of patients in any study group*.

**Table 4:** Adverse events affecting at least 5% of subjects receiving study drug.

**System organ class**	**Number (%) of patients**				
**Preferred term**	**All (*n* = 61)**	**QBECO-R (blinded) (*n* = 14)**	**QBECO-NR (open-label) (*n* = 16)**	**Placebo-R (blinded) (*n* = 9)**	**Placebo-NR (open-label) (*n* = 22)**
**Weeks 9–16**					
Any Adverse Event	29 (47.5)	5 (35.7)	7 (43.8)	5 (55.6)	12 (54.5)
Common Adverse Events^*^					
Abdominal pain	2 (3.3)	0 (0.0)	0 (0.0)	0 (0.0)	2 (9.1)
Abdominal tenderness	2 (3.3)	0 (0.0)	0 (0.0)	1 (11.1)	1 (4.5)
Abnormal feces	1 (1.6)	1 (7.1)	0 (0.0)	0 (0.0)	0 (0.0)
Crohn's disease	2 (3.3)	0 (0.0)	1 (6.3)	0 (0.0)	1 (4.5)
Haematochezia	1 (1.6)	1 (7.1)	0 (0.0)	0 (0.0)	0 (0.0)
Hemorrhoids	1 (1.6)	0 (0.0)	0 (0.0)	1 (11.1)	0 (0.0)
Large intestine perforation	1 (1.6)	0 (0.0)	1 (6.3)	0 (0.0)	0 (0.0)
Nausea	2 (3.3)	0 (0.0)	1 (6.3)	0 (0.0)	1 (4.5)
Paraesthesia oral	1 (1.6)	0 (0.0)	1 (6.3)	0 (0.0)	0 (0.0)
Vomiting	2 (3.3)	1 (7.1)	1 (6.3)	0 (0.0)	0 (0.0)
Chest discomfort	1 (1.6)	0 (0.0)	1 (6.3)	0 (0.0)	0 (0.0)
Fatigue	4 (6.6)	0 (0.0)	2 (12.5)	1 (11.1)	1 (4.5)
Influenza like illness	10 (16.4)	1 (7.1)	3 (18.8)	0 (0.0)	6 (27.3)
Injection site bruising	1 (1.6)	0 (0.0)	0 (0.0)	1 (11.1)	0 (0.0)
Pain	1 (1.6)	1 (7.1)	0 (0.0)	0 (0.0)	0 (0.0)
Pyrexia	4 (6.6)	1 (7.1)	1 (6.3)	1 (11.1)	1 (4.5)
Hepatic lesion	1 (1.6)	0 (0.0)	1 (6.3)	0 (0.0)	0 (0.0)
Hepatitis	1 (1.6)	0 (0.0)	1 (6.3)	0 (0.0)	0 (0.0)
Candidiasis	1 (1.6)	0 (0.0)	1 (6.3)	0 (0.0)	0 (0.0)
Folliculitis	1 (1.6)	0 (0.0)	1 (6.3)	0 (0.0)	0 (0.0)
Contusion	1 (1.6)	0 (0.0)	1 (6.3)	0 (0.0)	0 (0.0)
Exposure to toxic agent	1 (1.6)	0 (0.0)	0 (0.0)	1 (11.1)	0 (0.0)
C-reactive protein increased	1 (1.6)	0 (0.0)	1 (6.3)	0 (0.0)	0 (0.0)
Decreased appetite	1 (1.6)	0 (0.0)	1 (6.3)	0 (0.0)	0 (0.0)
Dehydration	1 (1.6)	1 (7.1)	0 (0.0)	0 (0.0)	0 (0.0)
Back pain	1 (1.6)	1 (7.1)	0 (0.0)	0 (0.0)	0 (0.0)
Groin pain	1 (1.6)	1 (7.1)	0 (0.0)	0 (0.0)	0 (0.0)
Myalgia	2 (3.3)	1 (7.1)	1 (6.3)	0 (0.0)	0 (0.0)
Headache	5 (8.2)	0 (0.0)	2 (12.5)	0 (0.0)	3 (13.6)
Tremor	1 (1.6)	1 (7.1)	0 (0.0)	0 (0.0)	0 (0.0)
Libido decreased	1 (1.6)	0 (0.0)	0 (0.0)	1 (11.1)	0 (0.0)
Renal failure	1 (1.6)	0 (0.0)	1 (6.3)	0 (0.0)	0 (0.0)
Pelvic pain	1 (1.6)	1 (7.1)	0 (0.0)	0 (0.0)	0 (0.0)
Cough	1 (1.6)	0 (0.0)	1 (6.3)	0 (0.0)	0 (0.0)
Oropharyngeal pain	1 (1.6)	0 (0.0)	1 (6.3)	0 (0.0)	0 (0.0)
Rhinorrhoea	1 (1.6)	0 (0.0)	1 (6.3)	0 (0.0)	0 (0.0)
Acne	1 (1.6)	0 (0.0)	0 (0.0)	1 (11.1)	0 (0.0)
Erythema	1 (1.6)	0 (0.0)	1 (6.3)	0 (0.0)	0 (0.0)
Hot flush	1 (1.6)	1 (7.1)	0 (0.0)	0 (0.0)	0 (0.0)
Hypotension	1 (1.6)	1 (7.1)	0 (0.0)	0 (0.0)	0 (0.0)

* *Common adverse events were defined as events occurring in at least 5% of patients in any study group. QBECO-R, responders to QBECO treatment stayed blinded on QBECO for weeks 9–16; QBECO-NR, non-responders to QBECO treatment went on open-label QBECO for weeks 9–16; Placebo-R, responders to Placebo treatment who stayed blinded on Placebo for weeks 9–16; Placebo-NR, non-responders to Placebo treatment went on open-label QBECO for weeks 9–16*.

Severe Adverse Events (SAE) were uncommon with 6.4 and 2.3% of all reported events reported as Grade 3 for those receiving QBECO and placebo, respectively. Seven of these eight (87.5%) individuals had past exposure to anti-TNFα therapies. Five of eight SAEs were considered to be unlikely related or unrelated to treatment, with 3 (fever/chills, liver/kidney issues, exacerbations of benign lung nodules) possibly related to treatment. The only SAE in an anti-TNFα-naïve subject was a *C. difficile* infection in an individual randomized to placebo. SAEs experienced during the course of the study and reasons for not completing the study are listed in Table 5.

**Table 5:** Serious adverse events experienced over the course of study.

**Weeks**	**ID**	**Treatment group**	**Verbatim term**	**Intensity**	**Effect on study drug**	**Relationship to study treatment**
1–8	11106	QBECO (blinded)	Mechanical bowel obstruction	Severe	Discontinued	Not related
1–8	11148	QBECO (blinded)	Fever and chills	Severe	Discontinued	Possibly
1–8	11154	Placebo (blinded)	*C. Difficile* infection	Severe	Discontinued	Not related
1–8	11168	QBECO (blinded)	Multiple pulmonary nodules (present before treatment; reactivation during study)	Severe	Discontinued	Possibly
9–16	11109	QBECO (open-label)	Hepatitis, renal failure	Severe	Discontinued	Possibly
9–16	11140	QBECO (open-label)	Perforated sigmoid	Severe	Discontinued	Not related
9–16	11144	QBECO (open-label)	Electrolyte abnormalities	Severe	Discontinued	Unlikely
9–16	11164	QBECO (open-label)	Exacerbation Crohn's disease	Moderate	Discontinued	Unlikely

#### Immune Biomarker Analysis

Forty-two serum immune factors were assessed at baseline, Week 8, 16, and 24. Interleukin-18 (IL-18) increased from baseline to Week 8 and 16 with QBECO treatment (median change 24 pg/mL, adjusted *p* = 0.066 at Week 8 and 56 pg/mL, adjusted *p* = 0.067 at Week 16), but not with placebo treatment. None of the serum cytokine biomarkers remained elevated at Week 24 (i.e., 8 weeks after stopping study treatment).

**Figure 4:**
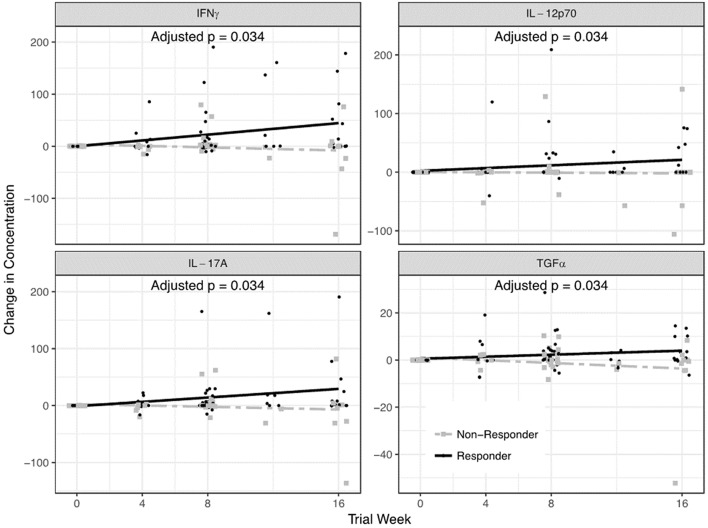
Serum cytokines that differed in QBECO responders and non-responders. A 42-plex cytokine/chemokine analysis was performed on serum. Four cytokines- IFNγ, IL-12p70, IL-17A, and TGFα- differentiated QBECO responders from non-responders over the study period after adjusting for multiple comparisons.

Among QBECO treated subjects, IFNγ, IL-12p70, IL-17A, and TGFα were significantly elevated in QBECO responders vs. QBECO non-responders (adjusted *p* = 0.037 for all) (Figure 4).

Week 8 clinical remission with QBECO treatment occurred more frequently in patients with lower baseline serum Eotaxin-1 levels (adjusted *p* = 0.0062) and IL-10 and IL-12p40 (*p* > 0.05 after correcting for multiple comparisons). This relationship between lower Eotaxin-1 levels and increased Week 8 remission was not found in placebo treated patients. These 3 cytokines tended to be higher in patients previously treated with TNFα inhibitors (Supplement Table 2), and in a longitudinal analysis, as performed with previous anti-TNFα agent use, patients with high baseline Eotaxin-1 levels responded equally well to QBECO treatment with a longer course of treatment (Supplement Figure 3).

CRP, an acute phase response protein upregulated in response to bacterial infections, and FCP, a cation-binding protein released by granulocytes in response to infection, were not anticipated to be reduced during active QBECO treatment given its mechanism of action. We assessed levels of these immune biomarkers leading up to Week 24 (when subjects were off study treatments). At Week 24, 44% of those who had been on QBECO from the beginning of the study, 42% of those who had switched to QBECO from placebo at Week 8, and 0% of those who were on placebo since the beginning had CRP levels < 5 mg/L (Supplement Table 3). Similarly, 35% of those who had been on QBECO from the beginning of the study, 18% of those who had switched to QBECO from placebo at Week 8, and 0% of those who were on placebo since the beginning had FCP levels of <250 ug/g (Supplement Table 4).

#### Genetic Associations With Response to QBECO

**Figure 5:**
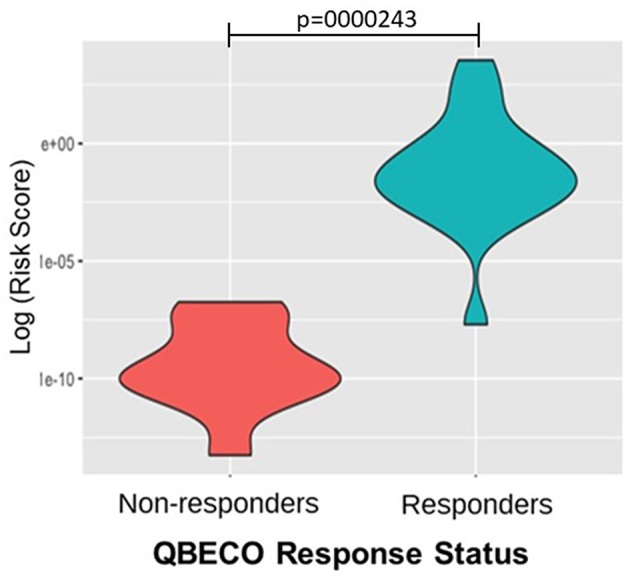
Gene risk score separates QBECO responders from non-responders. One hundred and thirteen inflammatory bowel disease (IBD)-related SNPs were included in computing the gene risk score for response to QBECO to assess the potential contribution of subjects' genetics to treatment outcome. The derived gene risk score could successfully distinguish QBECO responders from non-responders, *p* = 0.0000243.

One hundred and thirteen SNPs reported to be linked to IBD were analyzed for response to QBECO treatment. A gene risk score, which is a weighted value based on variation in multiple genetic loci, was computed to assess the ability to stratify subjects' response to QBECO. Figure 5 shows that the gene risk score could differentiate QBECO responders from non-responders in this cohort, *p* = 0.0000243. Supplement Table 5 lists the IBD-linked SNPs and their weighted contribution to the construction of the gene risk score.

## Discussion

In this proof-of-concept study assessing QBECO, a first-in-class microbial-based immunotherapy, for the treatment of CD, a greater reduction in disease was observed by Week 8 in subjects randomized to QBECO compared to placebo. For the pre-specified Week 8 primary analysis in this 68-patient study, the difference did not reach statistical significance, but secondary analyses suggest that the biological effect induced by QBECO may be of benefit to patients with moderate-to-severe CD and warrants further study.

Notably, patients with prior exposure to anti-TNFα agents, who are known to generally be more difficult to treat (22, 23), were less likely to respond to QBECO by Week 8. Due to unequal randomization, these patients were significantly more prevalent in the QBECO arm than in the placebo arm. However, subjects previously treated with TNFα inhibitors did experience symptom improvement with QBECO as treatment continued to Week 16, suggesting that a longer course of treatment may be required to achieve optimal results in these subjects. QBECO Week 8 clinical response, improvement and remission rates in anti-TNFα naïve patients compare favorably to those reported at similar time-points in recent Phase 3 trials with biologics such as vedolizumab and ustekinumab (22, 23).

The current treatment approach for CD largely targets the overzealous adaptive immune response to invading bacteria in the GI tract, but accumulating evidence suggests patients with CD have impaired or deficient innate immunity that predisposes to defective barrier function (3, 5, 7, 8). Identification of genetic variants linked to CD that associate with innate immune function lends support to the idea that innate immune insufficiency plays a role in disease pathophysiology, at least for a significant segment of those suffering from CD (5, 7). Serum cytokine analysis in this study demonstrated a QBECO-induced increase in IL-18, a cytokine known to promote phagocytosis and bacterial clearance (24). This corroborates our findings in experimental models of colitis in which colonic expression of IL-18 increased in response to QBECO treatment—resulting in marked improvements in gastrointestinal histopathology and barrier function (14). Other studies of colitis have shown a lack of IL-18 results in more severe disease (25), and the administration of IL-18 can reverse the phenotype (26). IL-18 with IL-12 acts on natural killer (NK) cells, γδ T cells and other “Th1” cells to stimulate the production of IFNγ (27, 28), which in turn acts on macrophages to further enhance phagocytosis, bacterial clearance and antigen presentation (24). CD patients who improved with QBECO treatment produced IL-18 and had increases in serum IFNγ, IL-12p70, and IL-17A levels, whereas, subjects deemed as QBECO non-responders did not show the same increases in these three cytokines. This may reflect an inability to launch a productive immune response to bacterial stimulation. The observed higher incidence of transient flu-like symptoms in QBECO treated subjects likely reflects this immune mobilization, and we believe it is part of QBECO's mechanism of action. Of note, CD patients who improved with QBECO treatment also had increases in their levels of TGFα, which has been reported to be reduced in diseased regions of the colon of patients with inflammatory bowel disease and increased in healthy regions (29).

Patients with lower baseline levels of Eotaxin-1, IL-10, and IL-12p40 were more likely to achieve response and remission with 8 weeks of QBECO treatment. Of note, these cytokines tended to be higher in those who had previously been treated with anti-TNFα therapy and may reflect greater immune dysregulation in patients (30–33). For such individuals, a longer course of QBECO treatment may be required to overcome the presence of greater immune dysfunction, as is suggested by the study's 16-week data showing that patients with higher baseline levels of these cytokines achieved more optimal responses with a longer duration of treatment.

A personalized approach to CD treatment has been elusive to date, possibly because current treatments focus on symptom management (3, 34, 35), rather than upstream biological processes predisposing CD symptoms. Subject genotype was found to differentiate QBECO responders from non-responders. Collectively, the genetic and cytokine findings of this study provide promise for personalized medicine in CD with QBECO, and they now need replication in larger cohorts.

This proof-of-concept study is limited by its small size, short treatment duration, lack of stratification for previous TNFα inhibitor use, and lack of endoscopic and histological assessment. The therapeutic paradigm has now moved from symptom-based assessment to objective measures of disease activity (36). QBECO treatment has shown endoscopic and histological improvement in moderate-to-severe ulcerative colitis (14) and now needs to be demonstrated in patients with CD.

In conclusion, QBECO warrants further study as a novel immunotherapy approach for the management of CD. This approach not only provides a new way of thinking about the treatment of the disease, but also sheds more light on the heterogeneity of CD pathogenesis. QBECO may be the optimal choice for those patients with disease characterized by innate immune dysfunction rather than other underlying etiologies. The data from this trial will inform the design of larger definitive Phase II trials, which will include evaluation of endoscopic and histological endpoints, assessment of the impact of prior TNFα inhibitor exposure on QBECO response, evaluation of patients over a longer treatment period, microbiome assessment, and confirmation of the genetic and immune biomarker findings.

## Ethics Statement

This was a Phase 1/2 randomized, double-blind, placebo-controlled study (NCT01809275; Health Canada approval 27-02-2013) for the treatment of moderate-to-severe CD. All study subjects provided written informed consent and the trial was conducted in compliance with the Declaration of Helsinki and Good Clinical Practice guidelines. The study protocol was approved by the institutional research ethics boards at the four study sites.

## Author Contributions

HG, SS, BB, and AC: study concept and design, analysis and interpretation of data, drafting and review of manuscript. SK, JP, MBa, GV, RF, RP, JA, JM, DMu, and DMc: analysis and interpretation of data, drafting manuscript, critical revision of manuscript, and acquisition of data. DT: statistical analysis of the trial. BK: statistical analysis of the genetic study. RG, JC, MBo, and JJ: acquisition of data, conduct of study, and patient engagement. All authors read and approved the final draft of the manuscript.

### Conflict of Interest Statement

SS, DT, RF, RP, DMu, BK, DMc, AC, and BB have served as consultants and/or advisors to Qu Biologics. SK, JP, JC, RG, MBo, MBa, JJ, and GV are (or were) employees of Qu Biologics. HG is the CEO and major shareholder of Qu Biologics. Qu Biologics owns patents across all the major markets (including U.S. Patent No. 8,980,279) relating to the use of Site Specific Immunomodulators derived from components of *E. coli* (QBECO) to treat inflammatory bowel disease. Qu Biologics has also filed patents for the use of immune and genetic biomarkers for the use of QBECO in patients with inflammatory bowel disease. In addition to the above, the following author affiliations are non-academic incorporated for-profit entities: Emmes Canada (DT) and Toronto Digestive Disease Associates, Inc. (JA). The remaining author declares that the research was conducted in the absence of any commercial or financial relationships that could be construed as a potential conflict of interest.
