# Supplementary material for: Novel Microbial-Based Immunotherapy Approach for Crohn's Disease
Source: Front Med (Lausanne). 2019 Jul 19;6:170. doi: 10.3389/fmed.2019.00170 (PMC6659126; doi:10.3389/fmed.2019.00170)
Supplement: Supplementary file 1 [file Data_Sheet_1.pdf]

Supplement Table 1: Mean Change in Crohn's Disease Activity Index (Per-Protocol Population)

<table>
<tr><td rowspan="2"></td><td colspan="4">Placebo</td><td colspan="4">QBECO</td></tr>
<tr><td colspan="2">N</td><td colspan="2">Mean Δ (SE)</td><td colspan="2">N</td><td colspan="2">Mean Δ (SE)</td></tr>
<tr><td>Week 8</td><td colspan="2">31</td><td colspan="2">-34 (14.6)</td><td colspan="2">27</td><td colspan="2">-84 (17.2)</td></tr>
<tr><td rowspan="2">Week 8
Mean Δ (SE)</td><td colspan="2">Placebo Responders (N=9)</td><td colspan="2">Placebo Non-Responders (N=22)</td><td colspan="2">QBECO Responders (N=13)</td><td colspan="2">QBECO Non-Responders (N=14)</td></tr>
<tr><td colspan="2">-140 (14.1) ↓</td><td colspan="2">10 (9.3)</td><td colspan="2">-162 (16.1) ↓</td><td colspan="2">-12 (9.5)</td></tr>
<tr><td rowspan="2"></td><td colspan="2">Placebo-Placebo (Blinded)</td><td colspan="2">Placebo-QBECO (Open-Label)</td><td colspan="2">QBECO-QBECO (Blinded)</td><td colspan="2">QBECO-QBECO (Open-Label)</td></tr>
<tr><td>N</td><td>Mean Δ (SE)</td><td>N</td><td>Mean Δ (SE)</td><td>N</td><td>Mean (SD)</td><td>N</td><td>Mean Δ (SE)</td></tr>
<tr><td>Week 16</td><td>9</td><td>-124 (21.6)</td><td>19</td><td>-100 (20.7)</td><td>13</td><td>-183 (23.3)</td><td>10</td><td>-70 (29.3)</td></tr>
</table>

Subjects randomized to either Placebo or QBECO treatment were evaluated at Week 8 (primary endpoint) for response to treatment using the Crohn's Disease Activity Index (CDAI). Response was defined as a decrease in CDAI ≥ 70 points. Those who responded to either blinded treatment, continued on blinded treatment to Week 16; non-responders were switched to open-label QBECO to Week 16. Statistics were not performed at Week 16 due to unequal distribution of subjects and small numbers in specific groups (such as those who stayed on Placebo through to Week 16; n<10). The data in this table is depicted in Figure 3 of the manuscript.

Supplement Table 2: Mean baseline serum levels of Eotaxin-1, IL-10 and IL-12p40 stratified by previous TNFα inhibitor exposure

	Previous anti-TNFα therapy	N*	Mean ± SD	Mean Difference ± SD	95% Confidence Interval of the Difference
**Eotaxin-1**	No	39	84 ± 43	-17 ± 12	(-41, 7)
	Yes	26	100 ± 54		
**IL-10**	No	28	6 ± 14	-5 ± 5	(-14, 4)
	Yes	17	11 ± 17		
**IL-12p40**	No	36	19 ± 67	-52 ± 36	(-123, 20)
	Yes	24	70 ± 198		

*20 reads from the IL-10 assay and 5 reads from the IL-12p40 were out of range of the assay or unreliable

Supplement Table 3: Proportion of subjects with C-reactive protein (CRP) levels of less than 5 mg/L at study weeks 12, 16, 20, and 24*

	QBECO-QBECO		Placebo-QBECO		Placebo-Placebo	
	Total	CRP<5	Total	CRP<5	Total	CRP<5
**Week**	**N**	**N (%)**	**N**	**N (%)**	**N**	**N (%)**
**12**	17	4 (23.5%)	19	4 (21.1%)	8	0 (0.0%)
**16**	18	7 (38.9%)	19	5 (26.3%)	8	0 (0.0%)
**20**	18	6 (33.3%)	19	5 (26.3%)	7	0 (0.0%)
**24**	18	8 (44.4%)	19	8 (42.1%)	8	0 (0.0%)

***QBECO-QBECO** refers to subjects who were randomized to QBECO and stayed on treatment to Week 16. **Placebo-QBECO** refers to subjects randomized to Placebo who switched to QBECO at Week 8 (through to Week 16). **Placebo-Placebo** refers to subjects randomized to Placebo who stayed on Placebo through to Week 16.

Note: Study treatment went to Week 16, subsequent weeks (i.e. weeks 20 and 24) were observational and subjects were off of all study treatments.

Supplement Table 4: Proportion of subjects with fecal calprotectin (FCP) levels below 100, 150 and 250 ug/g at study weeks 12, 16, 20, and 24*

	QBECO-QBECO			
Week	Total N	FCP<100 N (%)	FCP<150 N (%)	FCP<250 N (%)
**12**	21	4 (19.0%)	4 (19.0%)	7 (33.3%)
**16**	22	5 (22.7%)	6 (27.3%)	8 (36.4%)
**20**	18	3 (16.7%)	4 (22.2%)	5 (27.8%)
**24**	23	5 (21.7%)	6 (26.1%)	8 (34.8%)
	**Placebo-QBECO**			
**12**	14	3 (21.4%)	3 (21.4%)	3 (21.4%)
**16**	16	2 (12.5%)	2 (12.5%)	5 (31.2%)
**20**	17	3 (17.6%)	3 (17.6%)	5 (29.4%)
**24**	17	2 (11.8%)	2 (11.8%)	3 (17.6%)
	**Placebo-Placebo**			
**12**	8	0 (0.0%)	0 (0.0%)	1 (12.5%)
**16**	7	1 (14.3%)	1 (14.3%)	1 (14.3%)
**20**	6	0 (0.0%)	0 (0.0%)	0 (0.0%)
**24**	8	0 (0.0%)	0 (0.0%)	0 (0.0%)

***QBECO-QBECO** refers to subjects who were randomized to QBECO and stayed on treatment to Week 16. **Placebo-QBECO** refers to subjects randomized to Placebo who switched to QBECO at Week 8 (through to Week 16). **Placebo-Placebo** refers to subjects randomized to Placebo who stayed on Placebo through to Week 16.

Note: Study treatment went to Week 16, subsequent weeks (i.e. weeks 20 and 24) were observational and subjects were off of all study treatments.

Supplement Table 5: Inflammatory Bowel Disease (IBD)-associated single nucleotide polymorphisms (SNPs) used for the construction of the gene risk score for QBECO response

rsID	Risk Allele	OR
rs10065637	G	0.48
rs1016883	G	1.33
rs1042058	G	2.68
rs10521318	G	0.71
rs10758669	C	0.62
rs1077773	G	0.66
rs10781499	G	2.26
rs10865331	G	1.37
rs10896794	G	0.92
rs11054935	G	0.95
rs11083840	C	1.20
rs11150589	G	1.16
rs11168249	G	0.63
rs11209026	G	3.64
rs11583043	G	0.81
rs11672983	G	2.99
rs11739663	G	3.19
rs11742570	G	1.21
rs1182188	G	1.63
rs1260326	G	1.27
rs12778642	C	0.58
rs13204048	G	3.84
rs13277237	G	0.86
rs1517352	C	0.82
rs1569723	C	0.69
rs1654644	C	0.48
rs17085007	G	0.24
rs17119	G	1.60
rs17229285	G	0.56
rs1728918	G	0.61
rs1734907	G	3.56
rs17391694	G	12.00
rs1748195	G	1.06
rs17780256	C	0.76

rs1801274	G	2.94
rs1847472	C	0.93
rs1893217	G	0.90
rs194749	G	2.31
rs2024092	G	0.38
rs2111485	G	0.77
rs212388	G	3.19
rs2155219	C	1.79
rs2188962	G	1.15
rs2189234	C	1.59
rs2227551	C	0.95
rs2231884	G	1.14
rs2413583	G	5.67
rs2472649	G	1.87
rs2641348	G	0.44
rs2651244	G	0.59
rs26528	G	1.99
rs2816958	G	0.43
rs2823286	G	0.79
rs2836878	G	0.56
rs2930047	G	1.08
rs3024505	G	1.20
rs35320439	G	1.43
rs3742130	G	3.00
rs3764147	G	1.85
rs3766606	C	0.96
rs38904	G	1.15
rs395157	G	0.76
rs4243971	C	2.28
rs4409764	C	0.89
rs4692386	G	0.79
rs4728142	G	1.63
rs477515	G	0.94
rs4802307	C	2.49
rs4836519	G	2.37
rs483905	G	1.58
rs4976646	G	0.68
rs516246	G	1.87
rs559928	G	2.68

rs564349	G	1.44
rs566416	C	1.09
rs568617	G	1.14
rs6017342	C	0.82
rs6088765	C	0.38
rs616597	C	0.74
rs6426833	G	0.91
rs6651252	G	0.40
rs6667605	G	0.50
rs6863411	T	1.18
rs6920220	G	0.21
rs7097656	G	0.66
rs7134599	G	0.41
rs7210086	C	0.76
rs7236492	G	0.32
rs7240004	G	0.98
rs724016	G	1.25
rs7282490	G	0.70
rs7495132	G	1.33
rs7517810	G	19.83
rs7702331	G	1.48
rs7758080	G	0.95
rs864745	G	1.90
rs917997	G	0.38
rs921720	G	0.75
rs925255	G	1.49
rs9264942	G	1.23
rs9286879	G	0.05
rs9297145	C	0.67
rs9319943	G	0.71
rs941823	G	1.09
rs9491697	G	1.00
rs9847710	G	2.66
rs12199775	G	1.69
rs12654812	G	1.85
rs1292053	G	1.29
rs2227564	G	1.05
rs3197999	G	0.34
rs6074022	G	0.67

Supplement Figure 1. Difference in Crohn's Disease Activity Index (CDAI) change over time by anti-TNFα Exposure

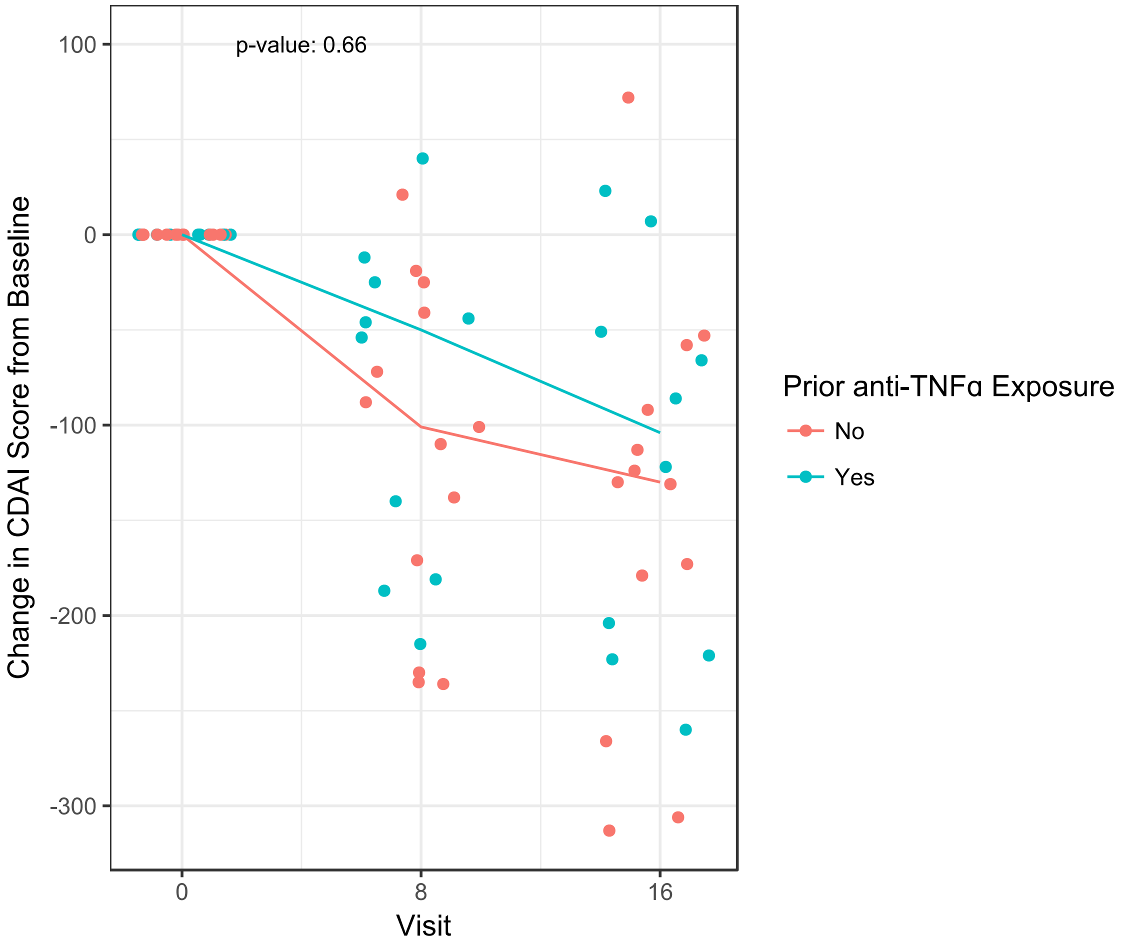

[1] Lines connect medians

[2] P-value is from a test of difference in trajectory beginning at Week 8 from a liner mixed-effect model of CDAI score over time.

Supplement Figure 2. Treatment outcomes adjusted for disease severity at baseline, use of concomitant immunosuppressive medication, disease duration and prior exposure to anti-TNFα agents

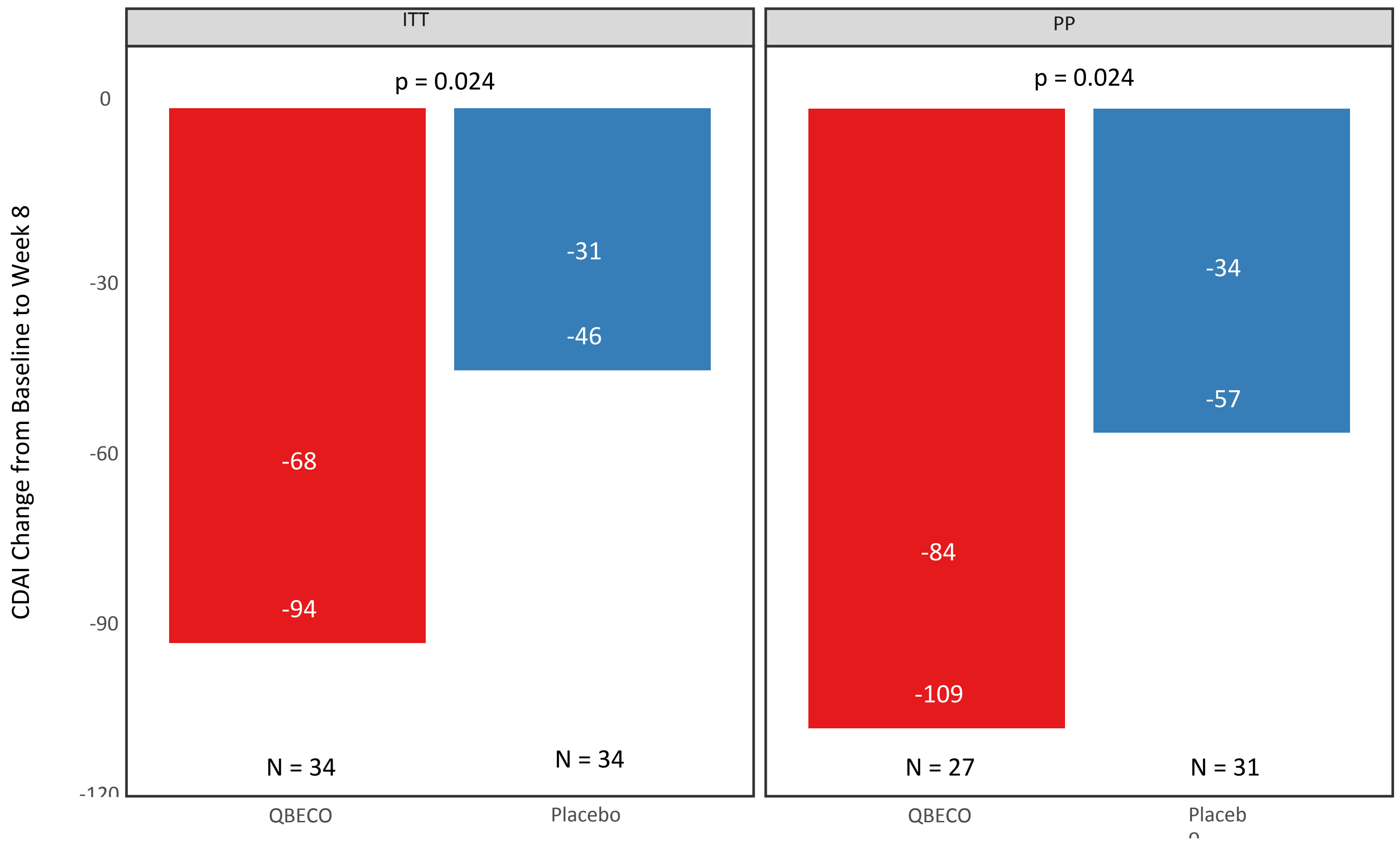

A regression analysis was performed to account for differences in baseline characteristics that may be important for treatment outcome (based on a reduction in the Crohn's Disease Activity Index; CDAI). The model indicated that the adjusted CDAI drop with QBECO treatment would be 94 points vs. 46 points for placebo in the intention to treat analysis (ITT). For the per protocol (PP) analysis, QBECO treatment would have a CDAI drop of 109 points vs 57 points for placebo.

Supplement Figure 3. Difference in Crohn's Disease Activity Index (CDAI) change over time by above or below median baseline Eotaxin-1

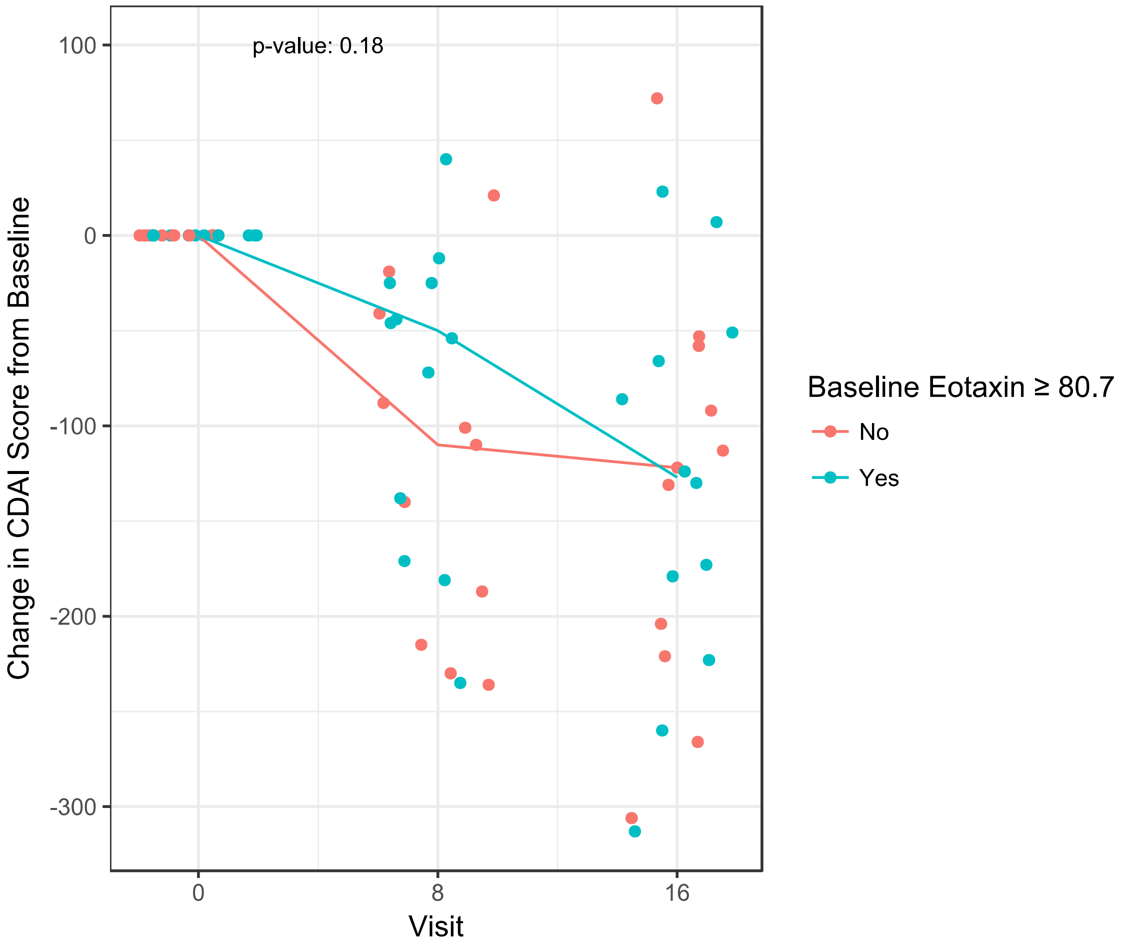

[1] Lines connect medians

[2] P-value is from a test of difference in trajectory beginning at Week 8 from a liner mixed-effect model of CDAI score over time.
